# Associations of Plasma Fatty Acid Patterns during Pregnancy with Respiratory and Allergy Outcomes at School Age

**DOI:** 10.3390/nu12103057

**Published:** 2020-10-07

**Authors:** Sara M. Mensink-Bout, Trudy Voortman, Marsela Dervishaj, Irwin K. M. Reiss, Johan C. De Jongste, Vincent W. V. Jaddoe, Liesbeth Duijts

**Affiliations:** 1 The Generation R Study Group, Erasmus MC, University Medical Center, 3000 CA Rotterdam, The Netherlands; s.mensink-bout@erasmusmc.nl (S.M.M.-B.); marseladervishaj@gmail.com (M.D.); v.jaddoe@erasmusmc.nl (V.W.V.J.); 2 Department of Pediatrics, Division of Respiratory Medicine and Allergology, Erasmus MC, University Medical Center, 3000 CA Rotterdam, The Netherlands; j.c.dejongste@erasmusmc.nl; 3 Department of Epidemiology, Erasmus MC, University Medical Center, 3000 CA Rotterdam, The Netherlands; trudy.voortman@erasmusmc.nl; 4 Department of Pediatrics, Division of Neonatology, Erasmus MC, University Medical Center, 3000 CA Rotterdam, The Netherlands; i.reiss@erasmusmc.nl; 5 Department of Pediatrics, Erasmus MC, University Medical Center, 3000 CA Rotterdam, The Netherlands

**Keywords:** fatty acids, child, inhalant allergic sensitization and allergy, pulmonary function, prospective cohort study

## Abstract

Fatty acids might play a role in asthma and allergy development as they can modulate immune responses. We examined among 4260 mother-child pairs participating in a population-based cohort the associations of maternal plasma fatty acid patterns during pregnancy with a child’s respiratory and allergy outcomes at school-age. In mid-pregnancy, 22 individual fatty acids were measured from maternal blood. Three patterns were previously identified by principal component analysis: A ‘high n-6 polyunsaturated fatty acid (PUFA)’, a ‘monounsaturated and saturated fatty acid’, and a ‘high n-3 PUFA’ pattern. At the age of 10 years, a child’s lung function was assessed by spirometry, current asthma and physician-diagnosed inhalant allergy by questionnaire, and inhalant allergic sensitization by skin prick tests. A higher ‘high n-6 PUFA’ pattern was associated with a higher forced expiratory volume in 1 s/forced vital capacity and forced expiratory flow after exhaling 75% of forced vital capacity (Z-score difference (95% CI) 0.04 (0, 0.07) and 0.04 (0.01, 0.07), respectively, per SD increase in the fatty acid pattern). We observed no associations of maternal fatty acid patterns with a child’s asthma or allergy outcomes. Our results showed limited associations of maternal patterns of high n-6 PUFA concentrations in pregnancy with a better lung function in school-aged children.

## 1. Introduction

Maternal diet during pregnancy has been related to respiratory and allergy outcomes in childhood [1]. Among many dietary factors, fatty acids may play an important role as they can modulate immune responses and might thereby influence the development of asthma and related diseases. It has been hypothesized that omega-6 (n-6) PUFAs (polyunsaturated fatty acids) stimulate the production of pro-inflammatory metabolites, including prostaglandins, thromboxanes, and leukotrienes, leading to allergic inflammation, whereas omega-3 (n-3) PUFAs can inhibit inflammation [2,3]. However, more recent studies suggest that the inflammatory effects of n-6 and n-3 PUFAs might differ between individual fatty acids and not be the same in all tissues [4]. Less emphasis has been put on saturated fatty acids (SFA) and monounsaturated fatty acids (MUFA), which might also have immunomodulating properties [5]. Previous intervention and observational studies have mainly focused on maternal PUFAs in relation to a child’s respiratory and allergy outcomes and provided inconsistent results [6,7,8,9,10]. A randomized controlled trial has shown that maternal supplementation with n-3 PUFAs during the third trimester of pregnancy reduces the risk of persistent wheeze or asthma in the children in the first 5 years of life with 7 percentage points as compared to children of mothers in the control group but has no effect on allergic sensitization [6]. We previously observed that higher total maternal n-6 PUFA concentrations during pregnancy were associated with a lower risk of asthma but not with airway inflammation [7]. Other observational studies have not found an association of maternal PUFA concentrations during pregnancy with asthma, lung function, or allergic sensitization in children [8,9,10]. However, these studies have been mainly performed among children in early childhood, while symptoms may arise at later ages. Furthermore, fatty acids interrelate with each other, and these synergistic or additive effects may be missed by previous studies that have examined fatty acids individually or in groups [11]. We aimed to overcome this by using fatty acid patterns, as this approach takes interrelations into account [12].

Therefore, our aim was to examine among 4260 children and their mothers participating in a population-based cohort study the associations of maternal plasma fatty acids patterns during pregnancy with a child’s lung function, asthma, inhalant allergic sensitization, and physician-diagnosed inhalant allergy at school-age.

## 2. Materials and Methods

### 2.1. Design and Cohort

This study was embedded in the Generation R Study, a population-based prospective cohort study from early fetal life onwards in Rotterdam, The Netherlands [13]. The study was approved by the Medical Ethical Committee of the Erasmus MC, University Medical Center in Rotterdam, The Netherlands (MEC-2012-165-NL40020.078.12). Written informed consent was obtained from the parents or legal representatives of all participating children. A total of 8663 women were enrolled before 25 weeks of gestation. Complete information on fatty acid profiles was available for 6997 women. Of this group, 6923 women gave birth to singleton live-born children, of whom 4260 had data on lung function, asthma, inhalant allergic sensitization, or physician-diagnosed inhalant allergy at the age of 10 years (Supplementary Figure S1).

### 2.2. Maternal Plasma Fatty Acid Patterns

Maternal venous blood samples for fatty acid composition analysis in plasma glycerophospholipids were drawn in the second trimester of pregnancy [14]. The samples were centrifuged in the regional laboratory, stored at −80 °C, and transported to the Division of Metabolic Diseases and Nutritional Medicine, Dr. von Hauner Children’s Hospital, Ludwig-Maximilians-University of Munich, Germany. As previously described, they were analyzed using gas chromatography, and the average coefficient of variation was 15.7% [15]. Information on the concentrations was available for 22 individual fatty acids. We previously applied principal component analysis on the weight percentage of the total fatty acids (wt%) of these 22 fatty acids [12]. Three patterns were identified and were named based on high factor loadings (≥0.20) for the respective fatty acids, describing how strongly each individual fatty acid contributes to each fatty acid pattern. The ‘high n-6 PUFA’ pattern was characterized by high factor loadings for myristic acid (lipid number, factor loading) (14:0, 0.23), palmitoleic acid (16:1n-7, 0.43), eicosenoic acid (20:1n-9, −0.25), eicosapentaenoic acid (20:5n-3, −0.33), docosahexaenoic acid (22:6n-3, −0.39), linoleic acid (18:2n-6, −0.45), γ-linolenic acid (18:3n-6, 0.53), dihomo-γ-linolenic acid (20:3n-6, 0.44), arachidonic acid (20:4n-6, 0.40), adrenic acid (22:4n-6, 0.85), osbond acid (22:5n-6, 0.82), and mead acid (20:3n-9, 0.53), the ‘MUFA and SFA’ by myristic acid (14:0, 0.30), palmitic acid (16:0, 0.88), margaric acid (17:0, −0.31), stearic acid (18:0, −0.84), palmitoleic acid (16:1n-7, 0.63), oleic acid (18:1n-9, 0.29), vaccenic acid (18:1n-7, 0.28), eicosenoic acid (20:1n-9, −0.31), linoleic acid (18:2n-6, −0.45), and dihomo-γ-linolenic acid (20:3n-6, 0.42), and the ‘high n-3 PUFA pattern’ by oleic acid (18:1n-9, 0.21), eicosapentaenoic acid (20:5n-3, 0.69), docosapentaenoic acid (22:5n-3, 0.59), docosahexaenoic acid (22:6n-3, 0.67), linoleic acid (18:2n-6, −0.67), eicosadienoic acid (20:2n-6, −0.69), arachidonic acid (20:4n-6, 0.30), and mead acid (20:3n-9, 0.37) (Supplementary Figure S2) [12].

### 2.3. School-Age Lung Function and Asthma and Allergy Outcomes

Children visited our research center at the age of 10 years. Lung function was assessed by spirometry (MS-Pneumo, Vyaire, Würzburg, Germany), according to the American Thoracic Society and European Respiratory Society (ATS/ERS) guidelines, and checked for acceptability and reproducibility [16]. Children who did not meet the reproducibility criteria of at least three curves with a ≤5% deviation in forced expiratory volume in 1 s (FEV_1_) and forced vital capacity (FVC), but with at least one technical acceptable curve with adequate reach and duration of the plateau (*n* = 281), were included for the current analyses, as including or excluding these children did not influence our results (data not shown). Lung function measures included FEV_1_, FVC, FEV_1_/FVC, and forced expiratory flow after exhaling 75% of FVC (FEF_75_) and were converted into sex-, height-, age-, and ethnicity-adjusted z-scores according to the Global Lung Initiative reference data [17]. Information on ever asthma, wheezing in the previous 12 months (no, yes less than 4 episodes, yes 4 or more episodes), and on physician-diagnosed inhalant allergy (no, yes) to pollen (hay fever), house dust mite, cat, or dog was obtained from questionnaires based on the International Study on Asthma and Allergy in Childhood (ISAAC) questionnaire [18]. Information on asthma medication use in the past 12 months was obtained during the research center visit. Current asthma (no, yes) was defined as reported ever physician-diagnosed asthma with either wheezing or the use of inhalant medication in the previous 12 months. Inhalant allergic sensitization (no, yes) to house dust mite, five-grass mixture, birch, cat, or dog (ALK-Abelló B.V., Almere, The Netherlands) was measured by skin prick tests using the scanned area method [19]. Skin responses were considered positive if the area of the wheel was ≥40% of that of the histamine response (i.e., histamine equivalent prick index area ≥0.40) [19].

### 2.4. Covariates

Questionnaires at enrollment and during pregnancy provided information on maternal age, pre-pregnancy body mass index, educational level, ethnic background, parity, history of asthma or atopy, smoking during pregnancy, folic acid supplementation, and total daily energy intake (kcal) in early pregnancy. Child’s sex, gestational age at birth (weeks), and birth weight (grams) were obtained from the midwife and hospital registries or questionnaires at birth. Postal questionnaires at age 6 and 12 months provided information about ever breastfeeding. At the age of 10 years, a child’s height and weight were measured, and age-adjusted childhood body mass index standard deviation scores were calculated [20].

### 2.5. Statistical Analysis

Characteristics of included and non-included children, children who did not have information on respiratory or allergy outcomes at the age of 10 years, were compared by using student’s *t*-test, Mann–Whitney U test, and chi-square test. Linear and logistic regression models were used to examine the associations of maternal fatty acid patterns with respiratory and allergy outcomes at the age of 10 years. In model 1 (basic model), we adjusted for gestational age at fatty acid measurement and child’s sex. In model 2 (confounder model), we additionally adjusted for maternal age, pre-pregnancy body mass index, educational level, ethnic background, parity, smoking during pregnancy, folic acid supplementation, total daily energy intake, and child’s breastfeeding. Confounders were included in our model based on the literature [21], if they were associated with both the exposure and the outcome, or if they changed the effect estimates with ≥10% compared to the basic model. We considered the confounder model as the main model.

When we observed a statistically significant association of maternal fatty acid patterns with child’s respiratory or allergy outcomes, we considered child’s growth factors as potential mediators and, therefore, additionally adjusted in model 3 for gestational age at birth and birth weight (early growth model) and in model 4 for child’s body mass index at the age of 10 years (childhood growth model). Potential non-linearity of the associations was evaluated by studying maternal fatty acid patterns in quartiles with the lowest quartile as the reference category. We additionally studied the associations of maternal fatty acid patterns with asthma severity as measured by the number of wheezing episodes by using multinomial regression models. Therefore, we created the groups ‘no current asthma’, ‘current asthma without wheezing episodes’, ‘current asthma with <4 wheezing episodes’, and ‘current asthma with ≥4 wheezing episodes’. Potential effect modifiers that we considered were maternal ethnic background, history of asthma or atopy, as women with atopy might have a different fatty acid metabolism, or child’s sex [21]. Additionally, we considered a child’s current asthma, inhalant allergic sensitization, and physician-diagnosed inhalant allergy as potential effect modifiers for the associations of maternal fatty acid patterns with a child’s lung function outcomes. We tested for effect modification by adding an interaction term to our main model and performed stratified analyses if the interaction term was significant (*p* < 0.10). Missing data for covariates were <20%, except for folic acid supplementation in early pregnancy (22.6%). To reduce bias and imprecision, we imputed missing data of the covariates with multiple imputations (m = 10) using the fully conditional specification method, and we reported the pooled effect estimates or odds ratios (OR) with their 95% confidence intervals (95% CI). Statistical analyses were performed using SPSS version 25.0 for Windows (IBM Corp., Armonk, NY, USA).

## 3. Results

### 3.1. Subject Characteristics

The characteristics of mothers and children included in the analyses are shown in Table 1. The prevalence of current asthma was 5.7% (*n* = 202), of inhalant allergic sensitization 32.7% (*n* = 1007), and of physician-diagnosed inhalant allergy 12.2% (*n* = 421). As compared with children in our analyses, those who were not included most importantly more often had mothers who had a lower education level, had less often a European ethnic background, and used less often folic acid supplementation (Supplementary Table S1).

### 3.2. Maternal Fatty Acid Patterns and Respiratory and Allergy Outcomes

In the basic model, we observed that a higher ‘high n-6 PUFA’ pattern was associated with a higher FEV_1_/FVC and FEF_75_. A higher ‘MUFA and SFA’ pattern was associated with a lower FEV_1_/FVC and FEF_75_. A higher ‘high n-3 PUFA’ pattern was associated with a lower FEV_1_, FEV_1_/FVC, and FEF_75_ (Table 2). After adjusting for confounders, only the associations of a 1 SD higher ‘high n-6 PUFA’ pattern with higher FEV_1_/FVC and FEF_75_ remained (Z-score difference (95% CI) 0.04 (0, 0.07) and 0.04 (0.01, 0.07), respectively) (Table 2). These associations did not attenuate after further adjustment for gestational age at birth and birth weight (Z-score difference (95% CI) 0.04 (0.01, 0.07) and 0.04 (0.01, 0.08), respectively) or child’s body mass index at the age of 10 years (Z-score difference (95% CI) 0.04 (0.01, 0.07) and 0.04 (0.01, 0.07), respectively), and, therefore, further mediation analyses were not performed. We did not observe any associations of maternal fatty acids patterns with current asthma, inhalant allergic sensitization, or physician-diagnosed inhalant allergy (Table 2).

When studying maternal fatty acid patterns in quartiles with the lowest quartile as a reference, we mainly observed similar directions of the results as in the linear models, and there were no indications for non-linear associations between fatty acid patterns and respiratory or allergy outcomes (Supplementary Table S2).

We did not find any associations of maternal fatty acid patterns with asthma severity divided into groups based on wheezing episodes (Supplementary Table S3).

We tested for interaction between the fatty acid patterns and maternal ethnic background, maternal asthma or atopy, and child’s sex. We only observed a significant interaction of a ‘high n-3 PUFA’ pattern with maternal ethnic background on the FEF_75_ (*p* = 0.01), and of a ‘high n-6 PUFA’ pattern with a maternal history of asthma or atopy on child’s current asthma (*p* = 0.10) and physician-diagnosed inhalant allergy (*p* = 0.07), and of a ‘high n-6 PUFA’ pattern with child’s sex on physician-diagnosed inhalant allergy (*p* = 0.05) (Supplementary Table S4). After stratification, none of the associations were statistically significant (Supplementary Table S4). When we tested for interaction between the fatty acid patterns and child’s current asthma, inhalant allergic sensitization, and inhalant allergy, we observed a significant interaction between a ‘high n-6 PUFA’ pattern and child’s current asthma on the association with FEF_75_ (*p* = 0.05), between a ‘high n-6 PUFA’ pattern and inhalant allergic sensitization on the associations with FEV_1_/FVC (*p* = 0.05) and FEF_75_ (*p* = 0.08), and between a ‘high n-6 PUFA’ pattern and physician-diagnosed inhalant allergy on the association with FVC (*p* = 0.07) (Supplementary Table S5). Stratification by current asthma and inhalant allergic sensitization showed similar directions for the associations of a ‘high n-6 PUFA’ pattern with FEV_1_/FVC and FEF_75_ in children with and without asthma or inhalant allergic sensitization, and stratification by physician-diagnosed inhalant allergy showed no consistent associations of a ‘high n-6 PUFA’ pattern with FVC (Supplementary Table S5).

## 4. Discussion

In this population-based prospective cohort study, we observed that a fatty acid pattern characterized by high concentrations of maternal n-6 PUFAs in pregnancy was associated with a higher FEV_1_/FVC and FEF_75_ in the children aged 10 years. Patterns characterized by high concentrations of most of the MUFAs or high concentrations of n-3 PUFAs were not consistently associated with a child’s lung function. We did not observe any associations of maternal fatty acid patterns with asthma or allergy outcomes in the children at school-age.

### 4.1. Comparison with Previous Studies

Only a single previous study examined the associations of maternal PUFA concentrations during pregnancy with a child’s lung function as measured by spirometry, showing no association of maternal PUFA concentrations during pregnancy with FEV_1_ in 6-year-old children, but other lung function outcomes were not taken into consideration [8]. We previously observed in younger children that higher maternal γ-linolenic acid (18:3n-6) and dihomo-γ-linolenic acid (20:3n-6) concentrations, both n-6 PUFAs, in pregnancy were associated with a lower airway resistance in the children at the age of 6 years, as measured by interrupter technique (Rint) [7]. We now additionally showed that a higher ‘high n-6 PUFA’ pattern during pregnancy, which includes both γ-linolenic acid and dihomo-γ-linolenic acid with high positive loadings, was associated with a better lung function at the age of 10 years, as reflected by FEV_1_/FVC and FEF_75_, which are more robust markers of airway obstruction.

Like most previous cohort studies on maternal fatty acid concentrations and an individual participant meta-analysis on the maternal intake of fish, being the main source of n-3 PUFAs, we did not find associations of maternal n-3 or n-6 PUFA’s during pregnancy with asthma, inhalant allergic sensitization, or physician-diagnosed inhalant allergy [8,9,10,22]. However, we previously observed in our cohort that a higher maternal total n-6 PUFA concentration was associated with a lower risk of asthma, whereas we did not find an association of a ‘high n-6 PUFA’ pattern with asthma in the current study [7]. Differences might be due to assessment of PUFAs in groups versus patterns, or the age at which asthma was assessed, at a younger age, as it is difficult to distinguish asthma from a viral-induced wheeze. A recent literature-based meta-analysis of randomized controlled trials mainly included studies with a follow-up until infancy and did not find a beneficial effect of n-3 PUFA supplementation in pregnancy on wheezing or asthma in the children [23]. Randomized controlled trials with a longer follow-up until school-age or young adulthood suggested that prenatal n-3 PUFA supplementation reduced the risk of asthma, although no consistent effect on lung function or allergy development was observed [6,24,25,26]. The beneficial effect of high-dose n-3 PUFA supplementation on asthma was the strongest in children of mothers with the lowest n-3 PUFA concentrations. Fish intake in The Netherlands and in the Generation R cohort is relatively low compared to other European countries [22,27]. It might be that the range of n-3 PUFA concentrations was too narrow in our cohort to detect a beneficial effect of higher maternal n-3 PUFA concentrations on a child’s asthma. Another explanation for the inconsistent results might be that the intervention trials were mainly performed in late pregnancy, whereas we assessed maternal n-3 PUFA concentrations in mid-pregnancy.

To our best knowledge, no previous studies examined the associations of maternal SFA and MUFA concentrations with a child’s respiratory or allergy outcomes at school-age. Although one study in Japanese mothers did not find an association of SFA or MUFA intake estimated from a questionnaire in pregnancy with wheezing at preschool age [28]. More studies on the effect of patterns of maternal PUFA, SFA, and MUFA concentrations at different time-points in pregnancy with a child’s respiratory and allergy outcomes at older ages are needed.

### 4.2. Interpretation of the Results

The observation of an association between high n-6 PUFA and better lung function at 10 years might be related to the immunological effects of a ‘high n-6 PUFA’ pattern. Arachidonic acid (20:4n-6), an important component in this pattern, can be converted into metabolites, including prostaglandin E2 (PGE_2_) [2]. Although PGE_2_ might enhance allergic inflammation, it seems to have an opposing and anti-inflammatory effect in the airway system [3]. Airway epithelium and smooth muscle are the main producers of PGE_2_, and PGE_2_ reduces bronchoconstriction, relaxes airway smooth muscle, and inhibits the recruitment of inflammatory cells and mast cell mediators [29,30]. These effects might contribute to a higher FEV_1_/FVC ratio and FEF_75_. The fatty acid metabolism and, consequently, the production of metabolites, including PGE_2_, might depend on genetic factors and differ between mothers or children with and without a history of atopic diseases [31]. However, although some *p*-values for interaction were significant, we did not observe differences in results among groups after stratification. We therefore considered that interaction between maternal ethnic background, maternal asthma or atopy, child’s sex, and child’s asthma or allergy and fatty acid patterns on the associations with lung function was minimally present. N-3 PUFAs might inhibit fetal T-helper 2 (Th2) responses through effects on the expression of genes and the production of pro-inflammatory eicosanoids, thereby lowering the risk of asthma and allergy [32]. During pregnancy, docosahexaenoic acid (22:6n-3) concentrations, one of the main n-3 PUFAs, increase until 18 weeks of gestation through mobilization from maternal stores, but concentrations decline to a deficiency in the third trimester [33]. This might explain the different observations between the randomized controlled trials in the third trimester and our study in mid-pregnancy.

The ‘MUFA and SFA’ pattern was characterized by high positive loadings of most of the MUFAs and of the SFAs—myristic acid (14:0) and palmitic acid (16:0)—but high negative loadings of the SFAs—margaric acid (17:0) and stearic acid (18:0). Different SFAs and MUFAs, such as palmitic acid and oleic acid, might have opposite inflammatory effects, which might explain a lack of association of this pattern with respiratory or allergy outcomes [34]. Furthermore, although PUFAs are transferred over the placenta, the fetus might synthesize MUFAs and SFAs de novo from glucose [35]. Maternal MUFA and SFA concentrations might, therefore, not fully reflect the fetal status of these fatty acids.

Despite the small effect estimates and absence of an association with clinical disease, our findings might be of importance from a developmental and population perspective. Fatty acid concentrations might partly depend on genetic and metabolic influences, although maternal diet might also play a role as 7.5% of the variation in the fatty acid patterns in our population was explained by differences in food intake [12]. Our results, therefore, suggest that future intervention studies should explore, in addition to the intake of maternal n-3 PUFAs, the intake of n-6 PUFAs and the interrelation between the different PUFA’s, as well as the role of genetic and metabolic influences in relation to respiratory and allergy outcomes in the children later in life.

### 4.3. Strengths and Limitations

This study was embedded in a large prospective population-based cohort study. We had detailed information available on maternal fatty acid concentrations during pregnancy and were able to examine fatty acids in patterns, which take the interrelation between individual fatty acids into account and avoid the chance of false-positive findings [12]. However, some limitations need to be mentioned. First, non-response analyses suggested a selection to a more healthy population, which would have led to biased estimates if the associations of interest were different between participants and non-participants. Second, although we measured a large number of individual PUFAs, they were only measured once in mid-pregnancy and, therefore, mainly reflect the dietary intake and metabolism of fatty acids in the preceding weeks but not in other periods of pregnancy [36]. Third, although we used validated and widely used questionnaires to assess asthma and physician-diagnosed inhalant allergy, information bias might have occurred, which might lead to under- or overestimations of the observed associations. Severe asthma in the general population is relatively uncommon. We therefore used an epidemiological approach based on the number of wheezing episodes, limiting the generalizability of the results. Lastly, although we adjusted for several sociodemographic and lifestyle-related confounders, we could not exclude residual confounding.

## 5. Conclusions

A maternal fatty acid pattern characterized by high levels of n-6 PUFAs in mid-pregnancy was associated with a better lung function, especially a higher FEV_1_/FVC ratio and FEF_75_, in school-aged children. We did not find consistent associations of fatty acid patterns with asthma or allergy outcomes. Future studies on the causality and clinical implications of the relation of a pattern of high n-6 PUFA concentrations in pregnancy with a better lung function in school-aged children are needed.

## Figures and Tables

**Table 1:** Characteristics of mothers and children included in the analysis.

	*n* = 4260
**Maternal Characteristics**	
Age, years	30.8 (4.8)
Pre-pregnancy body mass index (kg/m^2^) ^1^	22.6 (18.0–34.6)
Educational level, higher (%)	49.1 (2093)
Ethnic background, European (%)	64.8 (2760)
Parity, nullipara (%)	58.3 (2484)
History of asthma or atopy, yes (%)	38.5 (1640)
Smoking during pregnancy, yes (%)	25.7 (1096)
Folic acid supplementation, yes (%)	76.9 (3275)
Total daily energy intake (kcal)	2043 (553)
Gestational age at fatty acid measurement	20.7 (1.2)
Standard deviation scores for fatty acid patterns	
‘high n-6 PUFA’ pattern	−0.06 (0.98)
‘MUFA and SFA’ pattern	0.05 (0.97)
‘high n-3 PUFA’ pattern	0.08 (0.99)
**Child Characteristics**	
Sex, female (%)	50.3 (2142)
Gestational age at birth (weeks) ^1^	40.2 (35.7–42.4)
Birth weight (grams)	3443 (553)
Ever breastfeeding, yes (%)	92.0 (3920)
Body mass index (kg/m^2^)	17.6 (2.8)
FEV_1_ (z-score)	0.17 (0.97)
FVC (z-score)	0.20 (0.93)
FEV_1_/FVC (z-score)	−0.09 (0.96)
FEF_75_ (z-score)	0.04 (0.92)
Current asthma, yes (%)	5.7 (202)
Inhalant allergic sensitization, yes (%)	32.7 (1007)
Physician-diagnosed inhalant allergy, yes (%)	12.2 (421)

Values are means (SD), ^1^ medians (2.5–97.5th percentile), or valid percentages (absolute numbers), based on imputed data. Missing data on forced expiratory flow in 1 s (FEV_1_) (*n* = 533), forced vital capacity (FVC) (*n* = 533), FEV_1_/FVC ratio (*n* = 533), forced expiratory flow after exhaling 75% of FVC (FEF_75_) (*n* = 533), current asthma (*n* = 734), inhalant allergic sensitization (*n* = 1182), physician-diagnosed inhalant allergy (*n* = 819) were not imputed. Polyunsaturated fatty acid (PUFA), monounsaturated fatty acid (MUFA), saturated fatty acid (SFA).

**Table 2:** Associations of maternal fatty acid patterns with respiratory and allergy outcomes in children at the age of 10 years.

Fatty Acid Pattern (per SD)	FEV_1_ Z-Score Change (95% CI) *n* = 3727	FVC Z-Score Change (95% CI) *n* = 3727	FEV_1_/FVC Z-Score Change (95% CI) *n* = 3727	FEF_75_ Z-Score Change (95% CI) *n* = 3727	Current Asthma OR (95% CI) *n* = 3526	Inhalant Allergic Sensitization OR (95% CI) *n* = 3078	Inhalant Allergy OR (95% CI) *n* = 3441
**Model 1: Basic model**							
‘high n-6 PUFA’ pattern	0.01 (−0.03, 0.04)	−0.02 (−0.05, 0.01)	0.04 (0.01, 0.07) *	0.05 (0.02, 0.08) **	1.10 (0.95, 1.26)	0.95 (0.88, 1.02)	0.98 (0.88, 1.08)
‘MUFA and SFA’ pattern	−0.01 (−0.04, 0.03)	0.01 (−0.02, 0.04)	−0.04 (−0.07, −0.01) *	−0.04 (−0.07, −0.01) **	1.06 (0.91, 1.23)	0.98 (0.91, 1.06)	1.02 (0.91, 1.13)
‘high n-3 PUFA’ pattern	−0.04 (−0.07, −0) *	−0.02 (−0.05, 0.01)	−0.04 (−0.07, −0.01) *	−0.04 (−0.07, −0.02) **	0.94 (0.81, 1.09)	1.03 (0.95, 1.11)	0.98 (0.89, 1.09)
**Model 2: Confounder model**							
‘high n-6 PUFA’ pattern	0.01 (−0.03, 0.04)	−0.02 (−0.05, 0.02)	0.04 (0, 0.07) *	0.04 (0.01, 0.07) **	1.06 (0.91, 1.23)	0.95 (0.88, 1.03)	0.98 (0.88, 1.09)
‘MUFA and SFA’ pattern	0.01 (−0.03, 0.04)	0.01 (−0.01, 0.05)	−0.02 (−0.05, 0.02)	−0.01 (−0.04, 0.02)	1.12 (0.96, 1.30)	1.02 (0.94, 1.10)	1.07 (0.96,1.20)
‘high n-3 PUFA’ pattern	−0.02 (−0.05, 0.02)	−0.01 (−0.04, 0.02)	−0.01 (−0.05, 0.02)	−0.01 (−0.04, 0.03)	1.02 (0.87, 1.18)	1.08 (0.99, 1.17)	1.05 (0.94, 1.17)

Values are Z-score changes or odds ratios (OR) with 95% confidence interval (95% CI), derived from linear or logistic regression models per SD increase in the fatty acid patterns. Polyunsaturated fatty acid (PUFA), monounsaturated fatty acid (MUFA), saturated fatty acid (SFA), forced expiratory flow in 1 s (FEV_1_), forced vital capacity (FVC), forced expiratory flow after exhaling 75% of FVC (FEF_75_). Fatty acids are classified into three patterns, derived from the principal component analysis: ‘High n6 PUFA’ pattern, ‘MUFA and SFA’ pattern, and ‘High n-3 PUFA’ pattern. Model 1 (basic model) was adjusted for gestational age at fatty acid measurement and child’s sex. Model 2 (confounder model) was additionally adjusted for maternal age, pre-pregnancy body mass index, educational level, ethnic background, parity, smoking during pregnancy, folic acid supplementation, total daily energy intake, and child’s breastfeeding. * *p*-value < 0.05 and ** *p*-value < 0.01.

## Supplementary Materials

The following are available online at https://www.mdpi.com/2072-6643/12/10/3057/s1, Figure S1: Flow chart of participants included for analysis, Figure S2: Factor loadings of maternal individual fatty acids in fatty acid patterns, Table S1: Comparison of baseline characteristics between participants and non-participants in the analysis, Table S2: Associations of maternal fatty acid patterns in quartiles with respiratory and allergy outcomes in children at the age of 10 years, Table S3: Associations of maternal fatty acid patterns with asthma severity divided in groups based on wheezing episodes, Table S4: Associations of maternal fatty acid patterns with respiratory outcomes in children at the age of 10 years, stratified by maternal ethnic background, maternal history of asthma or atopy, and child’s sex, Table S5: Associations of maternal fatty acid patterns with respiratory outcomes in children at the age of 10 years, stratified by current asthma, inhalant allergic sensitization, and inhalant allergy.
