# Supplementary material for: Associations of Plasma Fatty Acid Patterns during Pregnancy with Respiratory and Allergy Outcomes at School Age
Source: Nutrients. 2020 Oct 7;12(10):3057. doi: 10.3390/nu12103057 (PMC7601105; doi:10.3390/nu12103057)
Supplement: Supplementary file 1 [file nutrients-12-03057-s001.pdf]

Supplementary material

Associations of plasma fatty acid patterns during pregnancy with respiratory and allergy outcomes at school age

Sara M. Mensink-Bout, MD[1,2], Trudy Voortman, PhD[3], Marsela Dervishaj, MD[1,2], Irwin K. Reiss, MD, PhD[4], Johan C. de Jongste, MD, PhD[2], Vincent W.V. Jaddoe, MD, PhD[1,5], Liesbeth Duijts, MD, PhD[2,4]

[1]The Generation R Study Group, Erasmus MC, University Medical Center, Rotterdam, the Netherlands.

[2]Department of Pediatrics, Division of Respiratory Medicine and Allergology, Erasmus MC, University Medical Center, Rotterdam, the Netherlands.

[3]Department of Epidemiology, Erasmus MC, University Medical Center, Rotterdam, the Netherlands.

[4]Department of Pediatrics, Division of Neonatology, Erasmus MC, University Medical Center, Rotterdam, the Netherlands.

[5]Department of Pediatrics; Erasmus MC, University Medical Center, Rotterdam, the Netherlands.

Figure S1. Flow chart of participants included for analysis

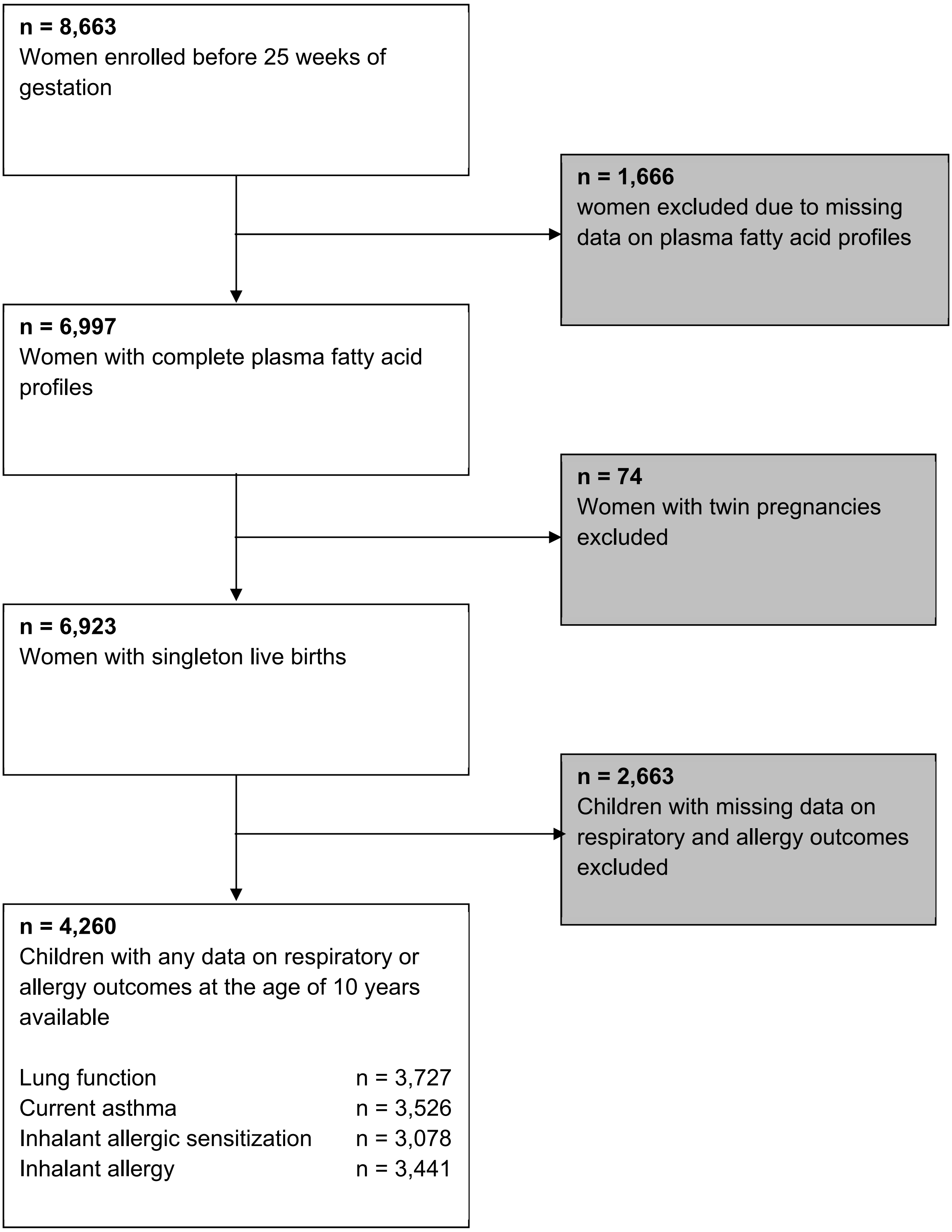

Figure S2. Factor loadings of maternal individual fatty acids in fatty acid patterns

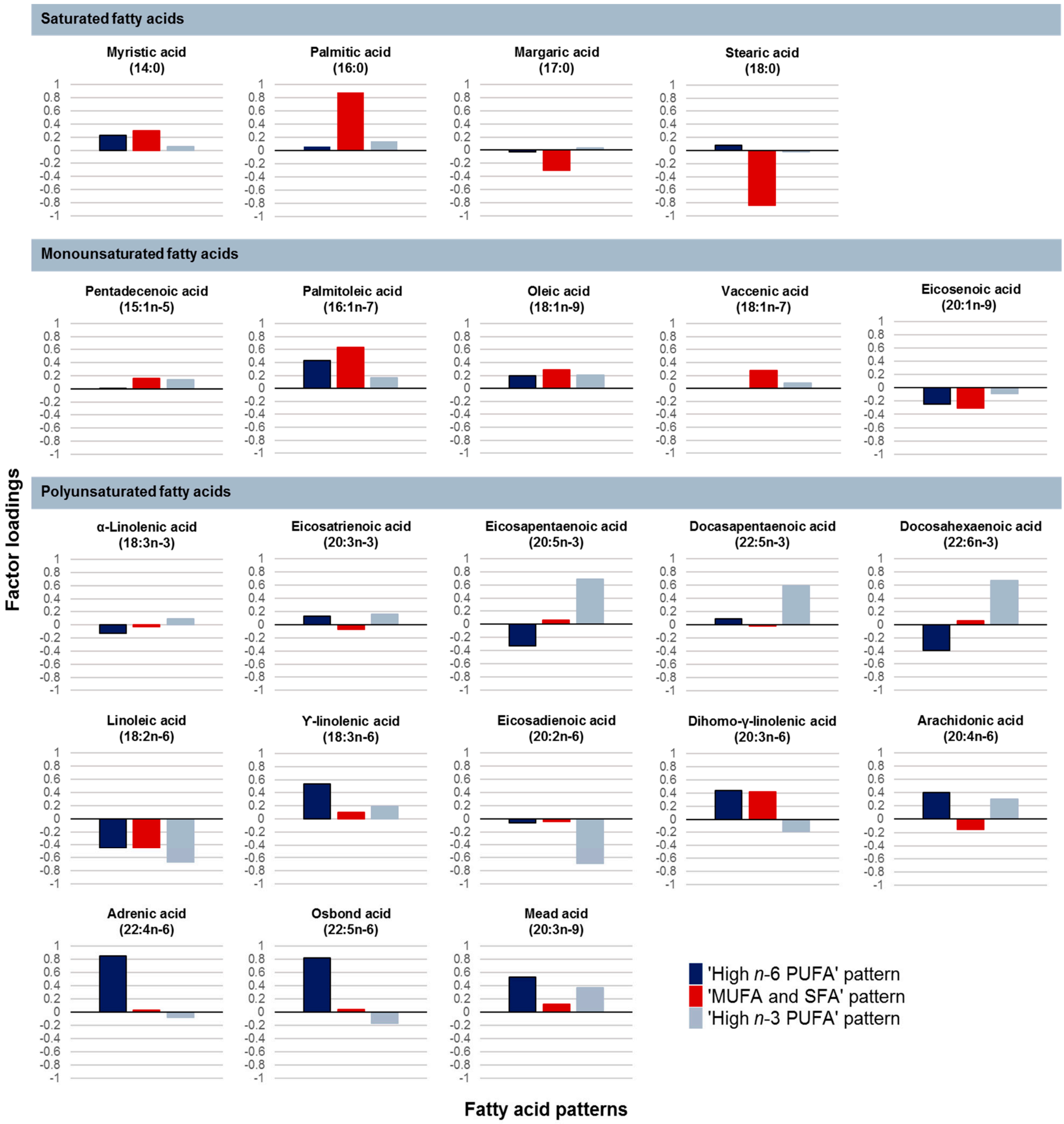

Polyunsaturated fatty acid (PUFA), monounsaturated fatty acid (MUFA), saturated fatty acid (SFA)

Table S1. Comparison of baseline characteristics between participants and non-participants in the analysis

	Participants<br><br>n= 4,260	Non-participants<br><br>n= 2,663	p-value for difference
**Maternal characteristics**			
Age, years	30.8 (4.8)	28.1 (5.4)	**<0.001**
Pre-pregnancy body mass index (kg/m^2)[1]	22.6 (18.0-34.6)	22.8 (17.6-36.1)	**0.027**
Educational level, higher (%)	50.3 (2,041)	28.5 (660)	**<0.001**
Ethnic background, European (%)	65.7 (2,752)	43.8 (1,061)	**<0.001**
Parity, nullipara (%)	58.5 (2,480)	52.0 (1,364)	**<0.001**
History of asthma or atopy, yes (%)	37.5 (1,651)	38.1 (109)	0.766
Smoking during pregnancy, yes (%)	24.4 (932)	25.2 (76)	0.763
Folic acid supplementation, yes (%)	78.8 (2,597)	58.8 (1,108)	**<0.001**
Total daily energy intake (kcal)	2057 (548)	2007 (603)	**0.003**
Gestational age at fatty acid measurement	20.7 (1.2)	20.7 (1.3)	0.424
**Child characteristics**			
Sex, female (%)	50.3 (2,242)	48.3 (1,286)	0.110
Gestational age at birth (weeks)[1]	40.1 (35.7- 42.4)	40.0 (35.0-42.3)	**<0.001**
Birth weight (grams)	3,444 (551)	3,378 (576)	**<0.001**
Ever breastfeeding, yes (%)	93.1 (3,235)	90.1 (247)	0.071
Body mass index (kg/m^2)	17.6 (2.8)	17.8 (2.7)	0.719

Values are means (SD), medians[1] (2.5-97.5th percentile) or valid percentages (absolute numbers) based on observed data. P-value for difference was calculated using Student's t-test for continuous normally distributed variables, Mann-Whitney U test for continuous not normally distributed variables, and chi-square test for categorical variables.

Table S2. Associations of maternal fatty acid patterns in quartiles with respiratory and allergy outcomes in children at the age of 10 years

Fatty acid pattern	FEV_1 Z-score change (95% CI) n = 3,727	FVC Z-score change (95% CI) n = 3,727	FEV_1/FVC Z-score change (95% CI) n = 3,727	FEF_{75} Z-score change (95% CI) n = 3,727	Current asthma OR (95% CI) n = 3,526	Inhalant allergic sensitization OR (95% CI) n = 3,078	Inhalant allergy OR (95% CI) n = 3,441
**'high n-6 PUFA' pattern**							
Quartile 1	Reference	Reference	Reference	Reference	Reference	Reference	Reference
Quartile 2	-0.05 (-0.14, 0.04)	-0.07 (-0.16, 0.01)	0.04 (-0.04, 0.13)	0.04 (-0.05, 0.12)	1.14 (0.75, 1.73)	0.94 (0.75, 1.16)	1.13 (0.85, 1.51)
Quartile 3	-0.01 (-0.10, 0.08)	-0.06 (-0.14, 0.03)	0.08 (-0.01, 0.16)	0.06 (-0.02, 0.15)	1.02 (0.67, 1.56)	1.06 (0.86, 1.32)	1.03 (0.77, 1.39)
Quartile 4	0.00 (-0.09, 0.09)	-0.04 (-0.13, 0.04)	0.07 (-0.02, 0.16)	**0.10 (0.02, 0.19)***	1.23 (0.81, 1.86)	0.81 (0.65, 1.01)	0.97 (0.71, 1.30)
**'MUFA and SFA' pattern**							
Quartile 1	Reference	Reference	Reference	Reference	Reference	Reference	Reference
Quartile 2	0.02 (-0.07, 0.11)	0.02 (-0.06, 0.11)	0.00 (-0.09, 0.09)	-0.01 (-0.09, 0.08)	1.02 (0.66, 1.57)	0.92 (0.74, 1.14)	0.90 (0.66, 1.22)
Quartile 3	0.05 (-0.04, 0.14)	0.06 (-0.02, 0.15)	-0.03 (-0.12, 0.06)	-0.00 (-0.08, 0.08)	1.18 (0.78, 1.79)	1.01 (0.82, 1.26)	1.04 (0.77, 1.40)
Quartile 4	0.03 (-0.06, 0.12)	0.05 (-0.04, 0.14)	-0.04 (-0.13, 0.05)	-0.02 (-0.10, 0.07)	1.33 (0.88, 2.02)	1.07 (0.86, 1.34)	1.19 (0.89, 1.59)

**'high n-3 PUFA' pattern**							
Quartile 1	Reference	Reference	Reference	Reference	Reference	Reference	Reference
Quartile 2	-0.07<br>(-0.16, 0.02)	-0.00<br>(-0.09, 0.08)	**-0.11**<br>**(-0.20, -0.02)***	-0.07<br>(-0.15, 0.02)	1.01<br>(0.67, 1.53)	0.94<br>(0.75, 1.17)	0.96<br>(0.70, 1.30)
Quartile 3	**-0.09**<br>**(-0.18, -0.00)***	-0.05<br>(-0.14, 0.04)	-0.06<br>(-0.15, 0.03)	-0.03<br>(-0.12, 0.05)	1.09<br>(0.73, 1.65)	1.10<br>(0.88, 1.37)	1.23<br>(0.92, 1.66)
Quartile 4	-0.07<br>(-0.16, 0.02)	-0.04<br>(-0.13, 0.05)	-0.04<br>(-0.13, 0.05)	-0.02<br>(-0.11, 0.06)	1.06<br>(0.69, 1.61)	1.20<br>(0.96, 1.51)	1.14<br>(0.84, 1.55)

Values are Z-score changes or odds ratios (OR) with 95% confidence interval (95% CI), derived from linear or logistic regression models per SD increase in the fatty acid patterns. Polyunsaturated fatty acid (PUFA), monounsaturated fatty acid (MUFA), saturated fatty acid (SFA), Forced Expiratory Flow in 1 second (FEV_1), Forced Vital Capacity (FVC), Forced Expiratory Flow after exhaling 75% of FVC (FEF_{75}). Fatty acids are classified into three patterns, derived from principal component analysis: 'High n6 PUFA' pattern, 'MUFA and SFA' pattern, and 'High n-3 PUFA' pattern. Models were adjusted for maternal age, pre-pregnancy body mass index, educational level, ethnic background, parity, smoking during pregnancy, folic acid supplementation, total daily energy intake, and gestational age at fatty acid measurement, and child's sex and breastfeeding. Bold indicates *p-value <0.05 and **p-value <0.01.

Table S3. Associations of maternal fatty acid patterns with asthma severity divided in groups based on wheezing episodes

Fatty acid pattern	No current asthma<br>Odds ratio<br>(95% CI)<br>n = 3,324	Current asthma without wheezing episodes<br>Odds ratio<br>(95% CI)<br>n = 98	Current asthma with <4 wheezing episodes<br>Odds ratio<br>(95% CI)<br>n = 78	Current asthma with ≥4 wheezing episodes<br>Odds ratio<br>(95% CI)<br>n = 24
**'high n-6 PUFA' pattern**	Reference	0.96 (0.77, 1.18)	1.22 (0.97, 1.54)	1.03 (0.68, 1.55)
**'MUFA and SFA' pattern**	Reference	1.03 (0.83, 1.28)	1.19 (0.93, 1.51)	1.15 (0.75, 1.76)
**'high n-3 PUFA' pattern**	Reference	1.00 (0.81, 1.24)	1.03 (0.81, 1.31)	1.07 (0.70, 1.62)

Values are odds ratios with 95% confidence interval (95% CI), derived from multinomial logistic regression models per SD increase in the fatty acid patterns. Polyunsaturated fatty acid (PUFA), monounsaturated fatty acid (MUFA), saturated fatty acid (SFA). Fatty acids are classified into three patterns, derived from principal component analysis: 'High n6 PUFA' pattern, 'MUFA and SFA' pattern, and 'High n-3 PUFA' pattern. Models were adjusted for maternal age, pre-pregnancy body mass index, educational level, ethnic background, parity, smoking during pregnancy, folic acid supplementation, total daily energy intake, and gestational age at fatty acid measurement, and child's sex and breastfeeding.

Table S4. Associations of maternal fatty acid patterns in with respiratory outcomes in children at the age of 10 years, stratified by maternal ethnic background, maternal history of asthma or atopy and child's sex

	**Maternal ethnic background**		**Maternal asthma or atopy**		**Child's sex**	
	**European**<br>**n = 2,760**	**Non-European**<br>**n = 1,500**	**No**<br>**n = 2,620**	**Yes**<br>**n = 1,640**	**Boy**<br>**n = 2,118**	**Girl**<br>**n = 2,142**
			**FEF_{75}**			
**'high n-3 PUFA' pattern**	-0.04<br>(-0.08, 0.00)	0.04<br>(-0.01, 0.10)	NA	NA	NA	NA
P-value for interaction	*0.01*					
			**Current asthma**			
**'high n-6 PUFA' pattern**	NA	NA	1.20<br>(0.97, 1.48)	0.94<br>(0.76, 1.17)	NA	NA
P-value for interaction			*0.10*			
			**Inhalant allergy**			
**'high n-6 PUFA' pattern**	NA	NA	1.09<br>(0.94, 1.26)	0.86<br>(0.72, 1.01)	1.06<br>(0.92, 1.23)	0.87<br>(0.74, 1.02)
P-value for interaction			*0.07*		*0.05*	

Values are Z-score changes with 95% confidence interval, derived from linear regression models per SD increase in the fatty acid patterns. Polyunsaturated fatty acid (PUFA), monounsaturated fatty acid (MUFA), saturated fatty acid (SFA), Forced Expiratory Flow after exhaling 75% of FVC (FEF_{75}). Fatty acids are classified into three patterns, derived from principal component analysis: 'High n6 PUFA' pattern, 'MUFA and SFA' pattern, and 'High n-3 PUFA' pattern. Models were adjusted for maternal age, pre-pregnancy body mass index, educational level, ethnic background, parity, smoking during pregnancy, folic acid supplementation, total daily energy intake, and gestational age at fatty acid measurement, and child's sex and breastfeeding. Not applicable (NA).

Table S5. Associations of maternal fatty acid patterns in with respiratory outcomes in children at the age of 10 years, stratified by current asthma, inhalant allergic sensitization and inhalant allergy

	Current asthma		Inhalant allergic sensitization		Inhalant allergy	
	**No** n = 3,324	**Yes** n = 202	**No** n = 2,071	**Yes** n = 1,007	**No** n = 3,020	**Yes** n = 421
			**FVC**			
**'high n-6 PUFA' pattern**	NA	NA	NA	NA	-0.03 (-0.06, 0.01)	0.09 (-0.02, 0.20)
P-value for interaction					*0.07*	
			**FEV_1/FVC**			
**'high n-6 PUFA' pattern**	NA	NA	**0.07 (0.03, 0.12)****	0.01 (-0.06, 0.08)	NA	NA
P-value for interaction			*0.05*			
			**FEF_{75}**			
**'high n-6 PUFA' pattern**	**0.04 (0.00, 0.07)***	0.16 (-0.00, 0.32)	**0.08 (0.03, 0.12)****	0.02 (-0.04, 0.09)	NA	NA
P-value for interaction	*0.05*		*0.08*			

Values are Z-score changes with 95% confidence interval, derived from linear regression models per SD increase in the fatty acid patterns. Polyunsaturated fatty acid (PUFA), Forced Expiratory Flow in 1 second (FEV_1), Forced Vital Capacity (FVC), Forced Expiratory Flow after exhaling 75% of FVC (FEF_{75}). Fatty acids are classified into three patterns, derived from principal component analysis: 'High n6 PUFA' pattern, 'MUFA and SFA' pattern, and 'High n-3 PUFA' pattern. Models were adjusted for maternal age, pre-pregnancy body mass index, educational level, ethnic background, parity, smoking during pregnancy, folic acid supplementation, total daily energy intake, and gestational age at fatty acid measurement, and child's sex and breastfeeding. Not applicable (NA). *p-value <0.05 and **p-value <0.01.
